# Assessment of a neuro-developmental screening tool in children in Bhutan

**DOI:** 10.12688/gatesopenres.13037.2

**Published:** 2019-09-11

**Authors:** Brian Wong, Sara Grundy, Lhab Tshering, Kinley Tshering, Farrah J. Mateen

**Affiliations:** 1 Department of Pediatric Neurology, Children's Hospital of Los Angeles, Los Angeles, CA, USA; 2 Department of Neurology, Massachusetts General Hospital, Boston, MA, 02114, USA; 3 Department of Psychiatry, Jigme Dorji Wangchuck National Referral Hospital, Thimphu, Bhutan; 4 Department of Pediatrics, Jigme Dorji Wangchuck National Referral Hospital, Thimphu, Bhutan; 5 Harvard Medical School, Boston, USA

**Keywords:** children, cognition, developmental delay, neurodevelopment, epidemiology

## Abstract

**Background:** Developmental screening tools are designed to fit the cultural context in which they are utilized, yet often find a wider international audience. This study evaluates the efficacy of one such tool, the Parental Evaluation of Developmental Status: Developmental Milestones (PEDS:DM), developed in the United States and tested in the lower income Asian country of Bhutan. We aimed to test the PEDS:DM instrument to measure neurodevelopmental delay in children in Bhutan.

**Methods:** In total, 96 community-dwelling Bhutanese children (3-7 years old) without diagnosed neurocognitive conditions were recruited from ambulatory clinics in urban Bhutan in 2016 as part of a larger study on retinal imaging and cognitive and growth parameters. Scoring was based on neurocognitive domains (gross and fine motor, receptive and expressive speech, self-help, social-emotional). Rates of failure (meant to indicate delay) within domains were calculated.

**Results:** Modifications of some standard questions were deemed necessary by the study staff to suit the cultural context, such as replacing kickball with football in a question regarding games played with rules to maintain local relevance. In a modified PEDS:DM test with these improvised modifications, the mean percentage of age-appropriate domains failed was 58.8% and the mean percent delay was 12.3% (range 0-41.4%, available in n=83). The highest prevalence of failures was 59.4% for receptive language and 76.3% for expressive language, much higher than the lowest rate of failure seen in self-help (5.4%).

**Conclusions:** The PEDS:DM requires further modifications and validation studies before it can be reliably implemented to assess developmental delay in children in Bhutan. In this pilot study, the rate of delay as reported by the PEDS:DM would be scored as markedly elevated, especially when compared to available epidemiologic studies in the region.

## Introduction

Neurodevelopmental delay is a common finding in United States pediatric practices, with the prevalence measured at 12–16% of all children ^1, 2^. For the purpose of referring children to appropriate early intervention services, various surveillance tools have been designed to help clinicians identify developmental delay along different cognitive domains. While in Western countries there exist robust protocols for surveiling children to identify developmental delay, there are few available data regarding the utility of such protocols in lower income countries. Nonetheless, it is estimated that the majority of children with developmental disabilities (up to 80%) reside in low- or middle-income countries ^3^.

A commonly utilized developmental screening tool in the U.S. is the Parents' Evaluation of Developmental Status: Developmental Milestones (PEDS:DM) ^4^. The survey is designed to assess developmental delay in children from birth to age 7–11 years across several cognitive domains: fine motor, gross motor, receptive language, expressive language, self-help, and social-emotional. While the PEDS:DM has been well validated in the United States where the instrument was constructed ^5, 6^, there are few studies confirming its validity across cultural contexts ^7^.

We aimed to assess the validity of the PEDS:DM survey in the lower-income country of Bhutan, a Himalayan country with no pediatric neurologists. Bhutan is typical of several lower-income countries where English is a language of school instruction and pediatric neurodevelopmental surveillance could be valuable, particularly if used by non-physician health care workers in the field.

## Methods

### Ethics approvals

The study was approved by the Research Ethics Board of Health, convened by the Bhutanese Ministry of Health (PO-2015-011) and the Institutional Review Boards at Massachusetts General Hospital (2015P000159) and Children’s Hospital of Los Angeles (CHLA-15-00539). Neurocognitive screening was implemented as part of a larger study to correlate cognitive functioning and retinal and other growth parameters ^8^. Informed consent (written, or via thumb print if parent had low literacy) was obtained from each subject’s parent or next of kin over the age of the majority.

### Location

The Kingdom of Bhutan is bordered by China and India. In 2014, the total expenditure on healthcare per capita was 89 USD, compared to 9402 USD in the USA ^9^. Subjects were recruited from the Jigme Dorji Wangchuck National Referral Hospital (JDWNRH) located in the capital city, Thimphu (population 138,736 in 2017) ^10^. Specialist care is available, but there are no pediatric neurologists or subspecialized developmental pediatricians. Patients presenting to the JDWNRH Department of Ophthalmology outpatient clinic for routine vision screening were recruited as subjects so as to recruit community-dwelling children who were not seeking care for neurodevelopmental or neurological issues directly. Dzongkha is the primary spoken language in Bhutan. English is taught in schools and is the primary written language ^11^.

### Enrollment

Recruitment took place in 2016. Inclusion criteria included all children aged three to seven years old. Children who did not have a parent present and who were unable to complete a retinal scan as part of a concurrent study ^8^ were excluded from the cognitive testing. Subjects were provided with reimbursement of travel expenses amounting to 500 Bhutanese Ngultrums (equivalent to approximately 12 USD). 

### Anthropometric characteristics

At the time of enrollment, each child’s height, weight, and head circumference were measured and recorded according to the methods of the World Health Organization ^12^. Z scores were calculated for the height, weight, and head circumference of each child by an independent scorer using normative data from the World Health Organization.

### Neurocognitive testing

The PEDS:DM survey was conducted on all eligible children by a U.S.-based pediatrician (BW) who was a fellow of pediatric neurology and native Bhutanese research staff (LT, KT). The survey assesses for cognitive development up to a maximum age limit, varying by task category. The domains and their corresponding upper age limits are: fine motor (6 years, 11 months), receptive language (7 years, 11 months), expressive language (6 years, 11 months), gross motor (4 years, 5 months), self-help (6 years, 11 months), and social-emotional (5 years, 5 months). Each domain includes questions of increasing difficulty correlated with specific ages. For each domain, each child was asked the question for their age, and then if incorrect, was asked the question for progressively younger age groups until he or she provided two consecutive answers correctly. Several questions were modified to provide greater cultural relevancy (see Extended data ^13^). 

The reading and math sections of the screen were not used because those questions were deemed irrelevant to the population. Examples include a math question which requires recognition of a penny. Bhutanese currency does not include coins. We also excluded a reading question that involved recognition of American “Exit” and “Caution” signs. To control for potential differences in parental literacy levels, surveys were conducted via interview by the pediatrician rather than having parents fill out a written survey. Subjects were only assessed in domains for which they were not over the maximum age limit for that domain. No other questions were excluded from the testing.

Although Dzongkha is the primary spoken language in Bhutan, the evaluations were conducted in English due to high levels of English proficiency in the majority of participants and parents ^11^. Forward and backward translation to Dzongkha was provided by a Bhutanese research assistant, who was available at every participant encounter, when requested.

With the assistance of the Bhutanese research assistant, some questions were mildly modified to be culturally relevant. For example, the social-emotional domain question, “can your child play games with rules, like board or card games, kickball, or hopscotch,” was altered to the example of “football” which is a more common childhood game in Bhutan.

### Scoring

Responses to questions were scored either based on parental reports of the child’s experience, or direct subject demonstration of the required task, as appropriate. For example, on a question in the fine motor domain, “can your child write any of the letters of the alphabet?” parents were asked to answer this question from their past experience of their child, and the subject was asked to perform the task. If there was a discrepancy between parental report and subject performance, then the score was determined by subject performance.

The PEDS:DM responses were scored as both an age-dependent *screen* (pass or fail for the age appropriate task) and *assessment* (percentage delay). For example, if the child was 6 years 5 months old and the highest level of a correctly answered question was for the level of 6 years 0 months, then she was scored as having failed the age-dependent *screen*. She would be considered 7.7% delayed ((1-(72 months/78 months))*100) in the *assessment*.

In addition to determining the number of children who fail (screen) and the percentage delay (assessment) within individual domains, a *screen composite score* (the number of domains failed divided by the number of age appropriate domains assessed) and *assessment composite score* (mean percent delay) were calculated across all domains per subject. This provided an overall assessment of delay for each child combining the domains assessed. To assess the patterns of failure across the study population, the rates of failure for each domain were assessed for all children as well as for each age group individually. Descriptions of each scoring metric are available in Table 1.

**Table 1.** Definitions of tool metrics on the PEDS:DM.

Metric	Subject(s) Assessed	Number of Developmental Domain(s) Assessed	Calculation
Domain specific screen	One child	Single	Pass or fail determination on the age-appropriate question for the domain
Screen composite score	One child	Multiple	Number domains failed/Number domains assessed
Domain specific assessment	One child	Single	(1-[Age level of the highest correctly answered question]/[child’s age*100])
Assessment composite score	One child	Multiple	Mean percentage delay across all domains assessed
Domain screen failure	All children	Single	Number of children who failed the domain/ Number of children assessed for the domain
Mean assessment percent delay	All children	Single	Mean percent delay for all children across a single domain

### Statistical analysis

Basic data analysis was descriptive and included means, proportions, and percentages for the variables of interest. Where sample sizes were reduced due to incomplete data, the analyzable subcohort sample size is provided. Scoring of the PEDS:DM test was performed using the provided testing manual by a study staff member who did not perform the cognitive or anthropometric testing of the subjects directly (SG). All calculations were performed using Stata 12 (StataCorp. 2011. *Stata Statistical Software: Release 12.* College Station, TX: StataCorp LP).

## Results

### Subject demographics and anthropometric characteristics

**Figure 1.**
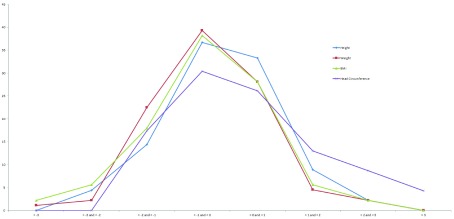
Distribution of weight, height, and body mass index for age, according to World Health Organization standardized growth curves. Y-axis is number of children (n=96). X-axis is z-score.

In total, 96 children (37 male) aged 3 years, 1 month to 7 years, 11 months old were enrolled. There were no refusals from parents to have their child participate. The mean age was 6 years 0 months old. 26 subjects were not yet enrolled in school ( Table 2 and Underlying data ^14^). Head circumference and height measurements followed a normal distribution. Weight and body mass index measurements skewed towards underweight (65.2% of subjects scored were under average weight) ( Figure 1).

**Table 2.** Participant characteristics at time of enrollment: age, education, medical comorbidities. (n=96 children).

	Male	Female	Total
**Total Participants** *	37	54	
Mean Age (months) ± SD	74 ± 14	70 ± 14	72 ± 14
Range (months)	37–95	37–94	37–95
**Duration of Schooling**			
No school	9 (24%)	15 (28%)	26 (28%)
>0 and <6 months	3 (8%)	1 (2%)	4 (4%)
>6 and <12 months	4 (11%)	7 (13%)	12 (13%)
>12 and <24 months	8 (22%)	15 (28%)	24 (26%)
>24 months	13 (35%)	14 (26%)	27 (29%)
**Medical Complications**	(n=36)	(n=52)	
Pregnancy complications (mother) (n=93)	1 (3%)	5 (10%)	6 (6%)
Pregnancy hospitalizations (mother) (n=87)	4 (12%)	8 (15%)	12 (13%)
Child medical problems (n=88)	5 (14%)	7 (14%)	12 (13%)

*Sex not recorded for 5 participants.

### PEDS:DM Scores

PEDS:DM *screen composite scores* were generated for 92 of 96 subjects, with possible scores ranging from 0 to 100. For the screen composite score, only an answer to the age appropriate question was required. Four children were missing data on the age appropriate question for their age. Of the remaining 92 children who could be scored, the average score was 41.2 points.

The *overall assessment score* determines the percentage of delay for a child across all domains. It required both the age-appropriate question and younger age groups’ questions to be answered correctly. Two questions in a row answered correctly were required to give a score. Of the 96 subjects, 13 were missing data that made the percent delay impossible to score. Of the remaining 83 children, the average score was 12.3 (range 0–41.4).

Domain screen failure rates were calculated for individual neurocognitive domains. The language domains contained the largest rates of failure on the PEDS:DM: 59.4% for receptive language, 76.3% for expressive language. The lowest rate of failure was seen in self-help (5.4%). 

The domain screen failure rates produced non-uniform results across childhood age groups. Percentage failure of the fine motor domain by age group ranged from 33.3–44.4%, with the striking exception of 0% for six-year-olds. Five-year-old children had the highest failure rate in receptive language (94.1%) compared to other ages, yet had the lowest failure rate in expressive language (64.7%). For the social-emotional domain, failure rate appeared to increase with age ( Table 3).

**Table 3.** Parental Evaluation of Developmental Status: Developmental Milestones (PEDS:DM) results. “Total positively screened” refers to the number of children by age group who screened positive in any single domain. The average screen composite score represents the number of domains failed over the number of domains assessed. The mean assessment is a measure of the mean percent delay across a single domain, while the average assessment composite score measures the mean percent delay across all of the domains assessed.

Age years [months]	3 [36-47]	4 [48-59]	5 [60-71]	6 [72-83]	7 [84-95]		Total
Number of subjects assessed	6	18	17	35	20		96
**Total positively screened by** **domain**							
Fine Motor (<95mo)	2 (33.3%)	8 (44.4%)	7 (41.2%)	0 (0.0%)	8 (40.0%)		25 (26.0%)
Receptive Language (<95mo)	1 (16.7%)	10 (55.6%)	16 (94.1%)	20 (57.1%)	10 (50.0%)		57 (59.4%)
Expressive Language (<83mo)	4 (66.7%)	15 (83.3%)	11 (64.7%)	28 (80.0%)	-		78 (76.3%)
Self-help (<83mo)	0 (0.0%)*	1 (5.6%)	1 (6.2%)*	2 (5.7%)	-		26 (5.4%)
Social-emotional (<65mo)	0 (0.0%)	6 (35.3%)*	6 (66.7%)**	-	-		76 (37.5%)
Gross Motor (<53mo)	1 (16.7%)	1 (16.7%)**	-	-	-		86 (16.7%)
**Average screen composite** **score by age group**	26.6 ± 8.8*	41.8 ± 21.8*	51.6 ± 21.2*	36.0 ± 17.6*	45.0 ± 39.4		41.2 ± 25.3*
	*n=5	*n=17, **n=6	*n=16, **n=9	*n=34			*n=92
							
**Mean assessment percent delay** **(% delay ± SD)**							
Fine Motor (<95mo)	4.5 ± 7.1	13.0 ± 13.0	8.6 ± 14.0	0 ± 0	2.9 ± 4.4		4.8 ± 9.7
Receptive Language (<95mo)	1.9 ± 4.7	18.8 ± 18.9	24.1 ± 12.0*	29.3 ± 12.7*	21.7 ± 22.1*		23.0 ± 17.1
Expressive Language (<83mo)	4.7 ± 4.2	19.6 ± 15.5	22.2 ± 9.1**	28.4 ± 12.0**	-		22.8 ± 13.5
Self-help (<83mo)	0 ± 0*	0.3 ± 1.3	0.3 ± 1.2**	1.3 ± 5.7	-		0.7 ± 4.0
Social-emotional (<65mo)	0 ± 0	5.7 ± 11.0*	6.1 ± 5.5***	-	-		4.7 ± 8.5
Gross Motor (<53mo)	2.9 ± 7.1	10.1 ± 16.0**	-	-	-		6.5 ± 12.4
							
**Average assessment composite** **score by age group**	2.3 ± 1.6	11.1 ± 10.0*	12.9 ± 6.3*	14.7 ± 5.8*	12.1 ± 11.5*		12.3 ± 8.6
	*n=5	*n=15, **n=6	*n=15, **n=16, ***n=9	*n=33, **n=32	*n=19		*n=83

The assessment composite score (percent delay) measured the degree of delay within particular domains. The score across all domains was 12.3% (range 0–41.4%, n=83). Similar to the screen composite scores, the language domains had the highest assessment composite scores. Within the language domains, the greatest delays were in the five- and six-year-old age groups (receptive language 24.1–29.3%, expressive language 22.2–28.4%). Relatively smaller total percent delays were observed in the motor (both fine and gross), social-emotional, and self-help domains, in a manner that roughly aligns with the failure rates for these domains in the domain specific screen.

## Discussion

The validation of culturally relevant screening tools provides a means for more accurate assessment of developmental delay in children in lower income countries as well as prevalence data on the extent of developmental delay in these locations. Here we evaluated the applicability of the PEDS:DM in Bhutan to determine its utility in this non-Western setting.

### Cultural differences leading to suboptimal performance

The exceedingly high rates of calculated delay in expressive and receptive language limit the ability to accurately interpret the other domains of the PEDS:DM test. A similar conclusion was reached in a comparative study conducted using the PEDS versus the PEDS:DM in a population of Thai children ^15^. That study reported a greater proportion of the subjects being classified with medium risk for developmental delay when compared to prior standardization studies in the United States. The authors postulated that local culture influenced certain responses of parental concern about their child’s development (such as expressing worry about a child being left-handed), resulting in an overestimation in the identification of actual delay. The high rates in the early school-year ages when Bhutanese children are increasingly exposed to the English language may also contribute to a perceived language delay, when truly there is none. Such a phenomenon has been reported consistently in bilingual children during early developmental stages of language, who are initially perceived to have expressive language delay in both languages, but eventually mature and have no language delay in either language ^16^.

The PEDS:DM was originally designed and intended for implementation in a Western patient population. Bhutanese cultural differences appeared to consistently invalidate certain questions of the test. For instance, for the self-help question, “Can your child get dressed by himself or herself?” almost all of the parents answered “no.” Bhutanese children are dressed in traditional outfits that are more complicated to put on compared to Western-style clothing, and thus children are usually unable to independently dress until an age much later than what was tested on the PEDS:DM. Children were often unable to correctly answer a question regarding irregular plural nouns (i.e. “tooth” and “teeth), suggesting such irregular English grammar structures are not introduced in the early school years, when most Bhutanese children are taught English. This may also account for the peak for delays in the assessment for expressive and receptive language occurring in five- and six-year old children, ages when subjects are first enrolled in formal schooling.

In school-age children, there was striking consistency in the manner that incorrect answers were provided. This may suggest a difference in academic curricula between the United States and Bhutan that led to suboptimal performance of Bhutanese children on an American-designed test. One such example may explain the high failure rate in receptive language among five-year-old children. In order to pass this domain, subjects had to identify at least three of the following body parts: shoulders, elbows, heels, ankles. Nearly all five-year-old children could correctly identify shoulders and elbows and very few could identify ankles and heels.

For six-year-old children to earn a “pass” on the fine motor domain, they had to correctly write three or more letters of the alphabet and draw a triangle with all lines connecting correctly. The abnormally high pass rate (100%) for this category amongst six-year-old children may be due to such skills being highly emphasized in school curriculum or at home by this age.

Our study gave credit to subjects who were able to demonstrate tasks, rather than accepting parents’ report on subjects’ ability. For example, subjects on one question were asked to recite the alphabet and would only be scored as “correct” if subjects could do so during the study visit. Subjects for the most part provided attempts at most questions. Valuing parental reporting over subject performance may have been a more representative assessment of the child’s abilities, as parents would more likely know the child’s skill performance in a natural environment.

### Comparison to the Two-Stage Child Disability Study Findings

The Two-Stage Child Disability Study was conducted by UNICEF and the National Statistics Bureau of Bhutan and was the largest recent country-wide screen of disability in Bhutan, including 3491 subjects ^17^. The study utilized a first-stage screen with the Ten Questions questionnaire (TQ) ^18^. Those who screened positive were then screened with the second stage: the Rapid Neuro-developmental Assessment (RNDA) for two to five year old children and the Rapid Functional Assessment (RFA) for five to nine year old children ^19^. The TQ, RNDA, and RFA were designed for use in low-income settings and focus on general function abilities, avoiding culture-specific skills (i.e. tying shoelaces). These tests were initially developed in large epidemiologic studies in Bangladesh, conducted by the Bangladesh Protibondhi Foundation, who provided these tests to the Two-Stage Child Disability Study ^17^. The tools have since been validated in other lower income settings on a country-wide scale in Jamaica and Pakistan ^20, 21^.

The Two Stage Child Disability Study provides a worthwhile comparison for our results. It reached a wider group of children geographically and with a much larger and population-based sample. While the authors of that study noted lower sensitivity and specificity of the first-stage TQ methodology when compared to similar studies conducted in other countries, they believed the final determination of *disability* from the second-stage screen to be accurate. Similar to our study, the Two-Stage Child Disability Study uses assessment tools that were created and validated elsewhere (Bangladesh), however the authors were careful to choose these assessments for their lack of testing of culture-specific skills.

As a rough point of comparison, when our data are compared to that of the Two-Stage Child Disability Study, we see that in all domains for which there was a direct comparison available, the percentage of disability measured by PEDS:DM exceeded that seen in the Two-Stage Study ( Table 4). However, with only one available comparable study, normative data in this population remain unclear.

**Table 4.** Comparison of PEDS:DM results to Two-Stage Child Disability Study in Bhutan.

Screen (failure rates)	PEDS Total	Two-Stage Study [% with disability]
	n=96	n=3491
Fine Motor (<95mo)	25 (26.0%)	199 (5.5%)
Receptive Language (<95mo)	57 (59.4%)	-
Expressive Language (<83mo)	78 (76.3%)	102 (2.3%)
Self-help (<83mo)	26 (5.4%)	-
Social-emotional (<65mo)	76 (37.5%)	-
Gross Motor (<53mo)	86 (16.7%)	86 (2.0%)

### Limitations and strengths

One limitation of our study is sampling from a more developed region of Western Bhutan. Our study does not generalize to the entire Bhutanese population which is more rural and remote, such as in the Eastern gewogs (Dzongkha term for a group of villages) where we did not enroll subjects. We suspect children suffering from significant disability in a lower income country could have accessibility issues, going to a hospital less often to accompany their parents for routine eye care or other appointments, and thus these children would not be well represented here. There is also the potential for selection bias, as subjects who could not complete the retinal scan as part of the larger study were not selected to complete the PEDS:DM. The retinal scan required children to sit still for several seconds; children with severe developmental delay would have had a more difficult time participating and would not be reflected in the PEDS:DM screening here. Furthermore, our subjects were chosen from children receiving routine vision examinations. This may bias our sample towards children whose parents have higher educational levels and socio-economic status, since these parents may be more likely to seek out such care. Parents seeking routine vision screening may also be more likely to have concerns regarding their child’s vision; if present, visual problems could be comorbid with developmental conditions.

While Dzongkha instructions were provided verbally, having written materials may also improve response accuracy during testing. Additionally, the PEDS:DM evaluation is designed as a screening test and therefore is intentionally sensitive in comparison to a confirmatory test such as was used in the second-stage of the Two Stage Child Disability study. This could be one of the reasons for the inflated rates of developmental delay measured in this study. It is also possible that subject performance anxiety increased the number of responses categorized as “incorrect.” If children are not accustomed to directly interacting with a healthcare provider during outpatient clinic visits, then requesting cognitive tasks may be overwhelming, leading to worse test outcomes. Finally, our small sample size overall and within each age group may contribute to the wide variations displayed between groups.

The female preponderance in our sample is considered a spurious finding. At the time of this study, the population ages 5 to 9 years old in Bhutan was 50.9% male and 49.1% female ^22^. To our knowledge there are no gender disadvantages at the pre-school or early school years for Bhutanese children. Nor are there known cultural considerations that may favor school age girls to receive routine medical care over boys.

Our study strengths include assessment of a community-dwelling pediatric population that is not well represented in the neurodevelopmental research literature. We are unaware of other studies of the PEDS:DM in Bhutan or neighboring regions. Children were evaluated by a senior fellow of pediatric neurology where none are otherwise available. We also provide a detailed assessment of how and likely why children may be erroneously *labeled as delayed* using a well-established surveillance tool in the U.S.A.

We demonstrate that the PEDS:DM may not be an appropriate developmental surveillance tool for use in children in Bhutan due to cultural differences in questions and required developmental testing tasks. When compared to the Bhutan Two-Stage Child Disability Study which recruited 3,491 Bhutanese children, our study of just less than 100 community-dwelling Bhutanese children using PEDS:DM categorized significantly more subjects with disability in one or more neurocognitive domains, at times more than half of the study population ( Table 4).

Diagnostic assessments that are sensitive to small differences in developmental delay are an important tool for early discovery and allow for early interventions in these cases. Low- and middle- income countries have the highest burden of developmental delay yet lack these important tools to recognize changes early when the largest differences on children’s outcomes can be made through early life intervention. The presumed lack of cross-cultural applicability of the PEDS:DM to a population in Bhutan shows the importance of developing pediatric neuro-developmental tools to better surveil children and determine accurate prevalence data in non-Western, non-English speaking countries. Locally-constructed developmental surveillance instruments such as the Angkor Hospital for Children Milestone Assessment Tool ^23^ utilized in Cambodia, the South Africa Road to Health Booklet ^224^ utilized in South Africa, and the Malawi Developmental Assessment Tool ^25^ utilized in Malawi, represent the promise of more valid, culture-specific tools on the horizon in other locations. While we do not suggest that tools such as PEDS:DM have no utility in this setting, we would advise culture-specific revisions of existing assessments before widespread implementation studies.

## Data availability

### Underlying data

Figshare: PEDS:DM Testing in Bhutan. https://doi.org/10.6084/m9.figshare.8226644 ^14^

This project contains the following underlying data:

Bhutan_fullTable_4June2019.xlsx (This is raw field data from 96 Bhutanese children undergoing the PEDS:DM evaluation. Cells where “N/A” or “not applicable” is entered reflect that the screening instrument is staged by the age of subject. Several subjects either were too young or failed to progress through the assessment, making more difficult questions no longer applicable to their testing.)

### Extended data

Figshare: Extended Data: Field Notes and Modifications to the PEDS:DM Test for Bhutanese Children. https://doi.org/10.6084/m9.figshare.8246282 ^13^

This project contains the following extended data:

- Modifications to PEDSDM_8Jun2019_10PM_FigShare.pdf (These are the field notes of the investigators on the modifications to the PEDS:DM in Bhutan. They are provided in text format and itemized by cognitive testing domain.)
