# Supplementary material for: Assessment of a neuro-developmental screening tool in children in Bhutan
Source: Gates Open Res. 2019 Sep 11;3:1496. Originally published 2019 Jun 20. [Version 2] doi: 10.12688/gatesopenres.13037.2 (PMC6694285; doi:10.12688/gatesopenres.13037.2)
Supplement: Peer review history

# Peer review: Reviewer response for version 2

**DOI:** 10.21956/gatesopenres.14192.r27825

**Published:** 2019-09-12

**Authors:** Philip Wilson

**Affiliations:** 1 Centre for Rural Health, University of Aberdeen, Inverness, UK

Thank you for responding to the reviews so comprehensively.  The article now reads very well and I am happy to give my approval.

I confirm that I have read this submission and believe that I have an appropriate level of expertise to confirm that it is of an acceptable scientific standard.

# Peer review: Reviewer response for version 1

**DOI:** 10.21956/gatesopenres.14150.r27618

**Published:** 2019-08-27

**Authors:** Issarapa Chunsuwan

**Affiliations:** 1 Department of Pediatrics, Faculty of Medicine, Developmental and Behavioral Pediatrics, Thammasat University (TU), Pathum Thani, Thailand

Thank you for giving me a chance to review this article. Research on the developmental screening in eastern countries and developing countries is interesting. Because of differences in context and culture. The use of screening tools which developed from the basis of western countries may have different results from from using that tool in the country of origin. This research shows that the use of PEDS: DM screening tools in Bhutan revealed different results from the United States.

 I have two main ideas about this research and some small suggestions.

 First, for the interpretation of the screening results using the PEDS: DM tools, it should be interpreted in the same way as the original, as this research aim to compare the use of this tool in Bhutan compare with original and other country. In table 1 which showed the definition of screening tool interpretation is quite different. The interpretation methods of the screening results by PEDS: DM tool, showed in table1, were different from the original. In the original, pass screening means that a child passes every item in a specific age range. Creating a new interpretation method made a comparison between the use of screening tools in Bhutan with in other countries difficult and confusing. The assessment composite score, which wants to show how slowly the child is behind the benchmark, seem not be the proper method for calculating the percentage of delay. The using of PEDS: DM assessment level screening should be more appropriate for evaluation. This version provides both age-equivalent scores, which can be used to determine percentage of delay ^1^.

 Second, In table 2, the author wanted to show the screening results that how many children in each age group have delays on each domain. Reporting the number of children with at least 1 delay in each age group may be more useful than showing the average screen composite score of age. Since the first reporting method can get values ​​that are compared with other research.

 For minor suggestion:

- Table 3, demographic data should presented before table 2
- Table 3, some numbers is confused, such as PEDs total number of gross motor domain was 86, while the total number of children age 3 + 4 who were screened in this domain was only 24
- The 2 staged study should be explained before showing results in table 3
- In the discussion about screening in Thailand, in that study, we use PEDS for screening (asked concern of parents about their child’s delay in each domain) and compared the results with PEDS: DM assessment level (assess children’s ability by researcher). We didn’t use the PEDS: DM for screening.

I agree with the author that the screening tools developed from one place should be adjusted to be used in another area, especially in the field of language. Due to the different language formats, the tools used to assess language development based on English ability cannot be used in other languages. Interestingly, that Bhutan uses English as the primary language in school, however development of the secondary language is differ from the native speaker, and the modification of language screening part should be done.

I confirm that I have read this submission and believe that I have an appropriate level of expertise to confirm that it is of an acceptable scientific standard.

## References

[1] : Parent Evaluation of Developmental Status (PEDS) in screening: The Thai experience. *Pediatr Int*.2016;58(12) : 10.1111/ped.13055 1277-1283 10.1111/ped.13055 27285278

# Peer review: Reviewer response for version 1

**DOI:** 10.21956/gatesopenres.14150.r27664

**Published:** 2019-08-12

**Authors:** Naila Z. Khan

**Affiliations:** 1 Clinical Neurosciences Center, Bangladesh Protibondhi Foundation, Dhaka, Bangladesh

The paper reads well. Reading the results, *without seeing the tables*, was easier. The tables are confusing in the discrepancies between the language of the column headings and the data provided. I have listed the confusions below.

Table 2:

1. The language of the first column headings and row numbers, as presented, don’t match. For example, in the second row it says ‘Screen (failure rates)’, while all the numbers entered within brackets (why brackets?) in that row, by age group, probably indicate number of children who failed. The first column should have read ‘number of screen positives’ and not ‘rates’. If rates are to be calculated then for each of the age groups (3,4,5,6,7) a ‘total number screened’ should be mentioned, and a percentage calculated. The next 8 rows calculate percentages of the 2 ^nd^ row ‘number of children’. Therefore, second row first column heading needs to be changed.
2. Last column: Two Stage Survey being used for comparison: Conversely, the second row number is the ‘Total number of children screened’. If we go by the 2 ^nd ^row heading of ‘Screen (failure rates)’, it does not make sense.
3. First column row 10, under Average Score Composite Score by Age Group, is confusing, as this row does not have a texted heading. How does this row differ from row 19, i.e. last row of the table, which also has number of children in brackets with asterisks? Please clarify, or redesign this table. I understand that each item in the first column has a different set of children seen. Still, too many asterisks above (row 10) and below (row 19) to follow.

 

Table 3:

 Rows and column heading are confusing. Repeats the same pattern as table 2.

1. Column one heading Age, years (96) simply repeats the Total Participants seen listed in row 2. Why not just say Mean Age in Years =/-SD (total=96) which is listed in row 4, and delete this row?
2. Years in School: This would mean to the reader ‘years of schooling’. However only total male/female numbers are given in the row. The next 5 rows under Years in School are even more confusing, as the age range goes from 0-24 months, i.e., below the pre-school age group. Otherwise, the reasons for their inclusion has to be made clear.
3. Sex not recorded asterisk below the table for 5 children: The asterisk should be in the 2 ^nd^ row after total number of participants, i.e., 96, as the numbers of male/female in that row don’t match the total.

My conclusions: The authors should re-design their tables, bearing in mind that each table should be self-explanatory. That is, they should not depend upon the readers reading the text to be able to understand. Headings, sub-headings, and sub sub-headings might be helpful. Just a suggestion.

I confirm that I have read this submission and believe that I have an appropriate level of expertise to confirm that it is of an acceptable scientific standard, however I have significant reservations, as outlined above.

# Response

**Published:** 2019-08-22

**Authors:** Farrah Mateen

“The paper reads well. Reading the results, *without seeing the tables*, was easier. The tables are confusing in the discrepancies between the language of the column headings and the data provided. I have listed the confusions below.”

Response: Thank you to the reviewer. We address the point by point comments in the revised text and in point form below.

Table 2:

“1.    The language of the first column headings and row numbers, as presented, don’t match. For example, in the second row it says ‘Screen (failure rates)’, while all the numbers entered within brackets (why brackets?) in that row, by age group, probably indicate number of children who failed. The first column should have read ‘number of screen positives’ and not ‘rates’. If rates are to be calculated then for each of the age groups (3,4,5,6,7) a ‘total number screened’ should be mentioned, and a percentage calculated. The next 8 rows calculate percentages of the 2 ^nd^ row ‘number of children’. Therefore, second row first column heading needs to be changed.”

Response: Adjusted first column heading to read “Total Positively Screened”. Added row entitled “number of subjects assessed” to better clarify how many subjects were assessed and how many subjects had screened positive. 

“2.    Last column: Two Stage Survey being used for comparison: Conversely, the second row number is the ‘Total number of children screened’. If we go by the 2 ^nd ^row heading of ‘Screen (failure rates)’, it does not make sense.”

Response: This column has been removed and placed into its own table (Table 4) for clarity.

“3.    First column row 10, under Average Score Composite Score by Age Group, is confusing, as this row does not have a texted heading. How does this row differ from row 19, i.e. last row of the table, which also has number of children in brackets with asterisks? Please clarify, or redesign this table. I understand that each item in the first column has a different set of children seen. Still, too many asterisks above (row 10) and below (row 19) to follow.”

Response: We now provide a heading that details out the various instruments used.

Table 3:

“Rows and column heading are confusing. Repeats the same pattern as table 2.

1.    Column one heading Age, years (96) simply repeats the Total Participants seen listed in row 2. Why not just say Mean Age in Years =/-SD (total=96) which is listed in row 4, and delete this row?”

Response: The mentioned row was deleted. We changed from measuring in years to months, as now seen in Table 2.

“2.    Years in School: This would mean to the reader ‘years of schooling’. However only total male/female numbers are given in the row. The next 5 rows under Years in School are even more confusing, as the age range goes from 0-24 months, i.e., below the pre-school age group. Otherwise, the reasons for their inclusion has to be made clear.”

Response: This may have been a source of confusion. We simply meant to say “duration of school attendance to date.”

“3.    Sex not recorded for the asterisk below the table for 5 children: The asterisk should be in the 2 ^nd^ row after total number of participants, i.e., 96, as the numbers of male/female in that row don’t match the total.”

Response: Asterisk position changed accordingly. 

“My conclusions: The authors should re-design their tables, bearing in mind that each table should be self-explanatory. That is, they should not depend upon the readers reading the text to be able to understand. Headings, sub-headings, and sub sub-headings might be helpful. Just a suggestion.”

Response: Thank you. We have also reordered and added a new table to clarify and allow the tables to stand separately from the paper. We thank the reviewer for the helpful comments and focus on the tables.

# Peer review: Reviewer response for version 1

**DOI:** 10.21956/gatesopenres.14150.r27420

**Published:** 2019-08-07

**Authors:** Charles R. Newton

**Affiliations:** 1 Kenya Medical Research Programme-Wellcome Trust Collaborative Programme, Kilifi, Kenya

This well-written paper describes the adaption and testing of the Parental Evaluation of the Developmental Status: Developmental Milestones (PEDS: DM) in 96 children (aged 3-8 years) who attended ambulatory clinics in urban Bhutan. The investigators found a high prevalence of developmental delay (59%), particularly in receptive language (59%) and expressive language (76%). They conclude (correctly in my view) that the PEDS: DM may not be culturally applicable to assess children in Bhutan.  

 It is not clear why the investigators choose to examine this tool in this setting when there are other screening tools e.g. Rapid Neurodevelopmental Assessment, Malawi Developmental Assessment Tool. A justification needs to be made more explicit.  

 There is little information provided on the adaption of the tool to the circumstances in Bhutan. There is a well-established process of adapting tools to different cultures and circumstances (van de Vijver and Tanzer, 2004 ^1^).

 The children were recruited from a clinic in a national referral hospital, and thus it is unclear how representative they are of the population. Most evaluations were conducted in English, suggesting that the population sampled were the more educated population. No data is provided on the socio-economic status of the parents or the maternal education.

 The influence of sex does not appear to have been taken into consideration during the analysis. 

 There was no information on the psychometric properties of the adapted PEDS: DM in Bhutan.

I confirm that I have read this submission and believe that I have an appropriate level of expertise to confirm that it is of an acceptable scientific standard.

## References

[1] : Bias and equivalence in cross-cultural assessment: an overview. *Revue Européenne de Psychologie Appliquée/European Review of Applied Psychology*.2004;54(2) : 10.1016/j.erap.2003.12.004 119-135 10.1016/j.erap.2003.12.004

# Response

**Published:** 2019-08-15

**Authors:** Farrah Mateen

“This well-written paper describes the adaption and testing of the Parental Evaluation of the Developmental Status: Developmental Milestones (PEDS: DM) in 96 children (aged 3-8 years) who attended ambulatory clinics in urban Bhutan. The investigators found a high prevalence of developmental delay (59%), particularly in receptive language (59%) and expressive language (76%). They conclude (correctly in my view) that the PEDS: DM may not be culturally applicable to assess children in Bhutan.  

It is not clear why the investigators choose to examine this tool in this setting when there are other screening tools e.g. Rapid Neurodevelopmental Assessment, Malawi Developmental Assessment Tool. A justification needs to be made more explicit.” 

*Thank you for making us aware of the other options available. We were not aware of such developmental screening tools at the time of this study. We now provide some examples of other relevant tools at the end of discussion.*

“There is little information provided on the adaption of the tool to the circumstances in Bhutan. There is a well-established process of adapting tools to different cultures and circumstances (van de Vijver and Tanzer, 2004 ^1^).

The children were recruited from an outpatient clinic in the national referral hospital. They represent a convenience sample only. It is unclear how representative they are of the full pediatric population. Most evaluations were conducted in English, suggesting that the population sampled were a more educated population. No data were queried on the socio-economic status of the parents or on the level of maternal education. *”*

*We plan to include this citation in the revised version.*

*We now discuss the potential for selection bias in our sample. You are correct that the sample is limited to a population living within travelling distance of the major urban center of the country. Since there is only one national hospital, it is possible that many children traveled long distances but more likely, these children have a marginally higher socio-economic status when compared to the rest of the country, which is primarily rural with less access to healthcare. Children were specifically selected as receiving routine vision screening, and maternal education likely play a part in which parents decide to seek such care. However, the entire health system is public, so all children would be seen free of direct costs.*

“The influence of sex does not appear to have been taken into consideration during the analysis. There was no information on the psychometric properties of the adapted PEDS: DM in Bhutan.”

*The following text will be included in results regarding gender distribution:*

The female preponderance in our sample is thought to be a spurious finding. The percentage of population of males to females ages 5-9 years at time of study was 50.9% male to 49.1% female. To our knowledge there are no gender disadvantages at the pre-school or early school years. Nor are there known cultural considerations that may favor school age females for receiving routine medical care over males.

# Peer review: Reviewer response for version 1

**DOI:** 10.21956/gatesopenres.14150.r27617

**Published:** 2019-08-02

**Authors:** Philip Wilson

**Affiliations:** 1 Centre for Rural Health, University of Aberdeen, Inverness, UK

Thank you for asking for an opinion on this interesting paper. It is a useful contribution to the world literature on developmental assessments and raises some important issues about the transfer of tools across cultures. The key finding is that, when using the PEDS: DM, a far higher proportion of children in Bhutan aged 3-7 years fail to meet expected milestones compared to children raised in the USA. The reasons for many such ‘failures’ lie in societal child-rearing practices described by the authors and are relatively easy to understand. Most would be relatively easy to remedy and the authors give valuable insights into how this might be achieved.

 I have two significant comments to make about the paper, and a few minor points which are largely stylistic.

1. The first major point is that it is not clear how representative of the general population of Bhutan (or indeed of the locality from which the sample was drawn) the 96 children are. Is the female preponderance a chance finding? Is the ophthalmological screening programme from which the children were drawn free of charge? Is it well attended, apart from by children with severe disabilities? Are parents with worries about visual abilities of their children more likely to attend? These sorts of concerns are known to be more common among parents of children with autism, for example. There is some discussion of these matters on page 7 in the Limitations and Strengths section, but a much more critical analysis of the potential for selection bias and its possible contribution to the findings is required. It is well understood that a completely representative sample is extremely difficult to obtain in lower income countries but readers will want to have some sort of idea about the proportion and nature of the potentially eligible population who would not have attended the clinic.
2. My second comment is about the use of the word screening. It is commonplace to refer to developmental screening, but no neurodevelopmental assessments have to date met standard WHO screening criteria - largely because there is little evidence that early intervention is any better than late intervention for most problems in most of the tested domains. Screening is most appropriately used for one-off assessments conducted in the context of an organised programme: ‘surveillance’ more closely reflects what is being done here. The authors might be interested in the discussion of this matter in a recent paper comparing child developmental assessments in five countries (Wilson *et al.*, 2018 ^1^). 

More minor points:

- I found Table 2 a little difficult to follow. The use of parentheses is confusing and the final column on the two-stage study does not make sense until the reader reaches the Discussion section.
- Ages would be best expressed in Months in both Table 2 and Table 3, and probably elsewhere in the text.
- The legend to Figure 1 requires more information including total n.
- The discussion about language assessment on page 6 should perhaps reference the literature on bilingualism (I assume that at least some of the children fall into this category). Young children reared in bilingual households commonly appear to have delayed language acquisition in each language but eventually achieve fluency in both.
- There are some minor typographical issues, such as in the first sentence of the Results section of the Abstract and the second sentence of the Strengths and Limitations section. 

I confirm that I have read this submission and believe that I have an appropriate level of expertise to confirm that it is of an acceptable scientific standard, however I have significant reservations, as outlined above.

## References

[1] : International variation in programmes for assessment of children's neurodevelopment in the community: Understanding disparate approaches to evaluation of motor, social, emotional, behavioural and cognitive function. *Scand J Public Health*.2018;46(8) : 10.1177/1403494818772211 805-816 10.1177/1403494818772211 29726749

# Response

**Published:** 2019-08-15

**Authors:** Farrah Mateen

“Thank you for asking for an opinion on this interesting paper. It is a useful contribution to the world literature on developmental assessments and raises some important issues about the transfer of tools across cultures. The key finding is that, when using the PEDS: DM, a far higher proportion of children in Bhutan aged 3-7 years fail to meet expected milestones compared to children raised in the USA. The reasons for many such ‘failures’ lie in societal child-rearing practices described by the authors and are relatively easy to understand. Most would be relatively easy to remedy and the authors give valuable insights into how this might be achieved.

I have two significant comments to make about the paper, and a few minor points which are largely stylistic.

1.    The first major point is that it is not clear how representative of the general population of Bhutan (or indeed of the locality from which the sample was drawn) the 96 children are. Is the female preponderance a chance finding? Is the ophthalmological screening programme from which the children were drawn free of charge? Is it well attended, apart from by children with severe disabilities? Are parents with worries about visual abilities of their children more likely to attend? These sorts of concerns are known to be more common among parents of children with autism, for example. There is some discussion of these matters on page 7 in the Limitations and Strengths section, but a much more critical analysis of the potential for selection bias and its possible contribution to the findings is required. It is well understood that a completely representative sample is extremely difficult to obtain in lower income countries but readers will want to have some sort of idea about the proportion and nature of the potentially eligible population who would not have attended the clinic.”

*We will include the following text in the discussion on limitations of study:*

Our study includes a convenience sample only. The referral hospital from which subjects were recruited was in the major urban area of the country. Subjects were chosen from those receiving routine vision screening, which likely may skew towards parents of a higher education level and socio-economic status, who would be assumed to be mostly likely to seek such form of child care. Those parents seeking routine vision screening may also be more likely to have pre-existing concerns about their child’s vision, which in turn may be associated with comorbid developmental conditions. 

Since the Bhutanese health care system is public and free for all Bhutanese citizens, the wealth gradient is not anticipated to be a major impact except for out of pocket costs to reach the hospital. These journeys to the main hospital can be costly in a mountainous country with difficult terrain but are culturally normal there. 

“2.    My second comment is about the use of the word screening. It is commonplace to refer to developmental screening, but no neurodevelopmental assessments have to date met standard WHO screening criteria - largely because there is little evidence that early intervention is any better than late intervention for most problems in most of the tested domains. Screening is most appropriately used for one-off assessments conducted in the context of an organised programme: ‘surveillance’ more closely reflects what is being done here. The authors might be interested in the discussion of this matter in a recent paper comparing child developmental assessments in five countries (Wilson *et al.*, 2018 ^1^).”

*Thank you for this observation. Will change* screening *to* surveillance *where appropriate.*

More minor points:

- I found Table 2 a little difficult to follow. The use of parentheses is confusing and the final column on the two-stage study does not make sense until the reader reaches the Discussion section. *Removed some extraneous parentheses around sample sizes*.
- Ages would be best expressed in Months in both Table 2 and Table 3, and probably elsewhere in the text. *Changed ages to months in table 2 and 3.*
- The legend to Figure 1 requires more information including total n. *Added total n to y-axis.*
- The discussion about language assessment on page 6 should perhaps reference the literature on bilingualism (I assume that at least some of the children fall into this category). Young children reared in bilingual households commonly appear to have delayed language acquisition in each language but eventually achieve fluency in both. *Thank you for this helpful observation. Added text on bilingualism in children to beginning of discussion.*
- There are some minor typographical issues, such as in the first sentence of the Results section of the Abstract and the second sentence of the Strengths and Limitations section. *These have been fixed. Thank you.*
